# Zinner Syndrome with Ectopic Ureter Emptying into Seminal Vesicle

**DOI:** 10.1155/2021/8834127

**Published:** 2021-01-25

**Authors:** M. Hevia Palacios, M. Álvarez-Maestro, J. Gómez Rivas, A. Aguilera Bazan, L. Martínez-Piñeiro

**Affiliations:** ^1^Member of Urology Department, Ramon y Cajal Hospital, Spain; ^2^Member of Urology Department, La Paz Universitary Hospital, Spain; ^3^Member of Urology Department, Clinico San Carlos Hospital, Spain; ^4^Section Chief, La Paz Universitary Hospital, Spain; ^5^Chief Urology Department, La Paz Universitary Hospital, Spain

## Abstract

A 66-year-old male patient in follow-up in the urology department for a non-muscle-invasive bladder cancer was detected by ultrasound to have absence of the left kidney and a cystic, multilobed image at the location of the seminal vesicle. Magnetic resonance imaging reveals left renal agenesis and the existence of multiple cysts in the ipsilateral seminal vesicle that reaches a size of 6.9 × 3.7 cm, as well as a ureteral remnant that opens into the seminal vesicle. The patient does not present urinary symptoms, neither pain with ejaculation nor hematuria. A triad of seminal vesicle cyst, ipsilateral renal agenesis, and ipsilateral ejaculatory duct obstruction is known as Zinner syndrome. Congenital anomalies of the seminal vesicles are rare; some of them are associated with malformations of the upper urinary system. Seminal vesicle cysts are associated with ipsilateral renal agenesis and ectopic or dysplastic ureter. Patients may remain asymptomatic and be diagnosed incidentally or may present with symptoms such as increased urinary frequency, dysuria, recurrent infections, pain with ejaculation, and perineal discomfort.

## 1. Introduction

Zinner syndrome, known as the triad of renal agenesis, cysts in the ipsilateral seminal vesicle, and ejaculatory duct obstruction, was described by A. Zinner in 1914 [1] and around 200 cases have been reported in the literature [2]. Congenital anomalies of the seminal vesicles are infrequent, most of them have cystic malformations and some are associated with malformations of the upper urinary system. The common embryological origin of the urinary and genital tracts (mesonephros or Wolf ducts and the ureteric bud) can produce, in the presence of an anomaly, an alteration in the development of both systems [3].

## 2. Case Report

A 66-year-old male patient with no comorbidities, ex-smoker, on follow-up for a non-muscle-invasive bladder cancer operated resulting in T1G2 in the pathological examination, is subsequently receiving treatment with BCG and without recurrences during follow-up. The patient remains asymptomatic and does not report pain with ejaculation or perineal discomfort.

Physical examination and blood tests, including kidney function and PSA, did not show any abnormalities. The digital examination revealed a fibroelastic prostate, without pathological findings. The patient did not develop fertility problems throughout his life, being able to have two healthy children.

**Figure 1:**
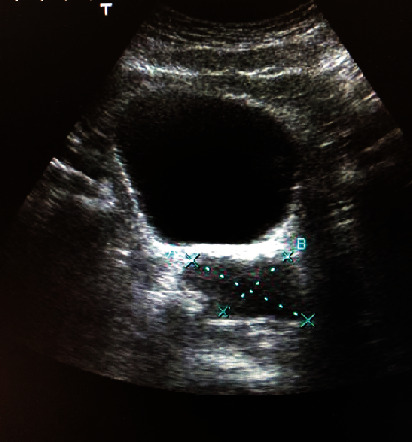
Abdominal ultrasound showing a cystic lesion close to the bladder.

In the initial evaluation ultrasound, the absence of the left kidney was detected, as well as a left posterolateral multilobed lesion on the location of the seminal vesicle, with cystic areas and echogenic content of approximately 7 × 3.5 cm (Figure 1).

Endoscopic examination reveals an absence of the left ureteral meatus, without other findings.

**Figure 2:**
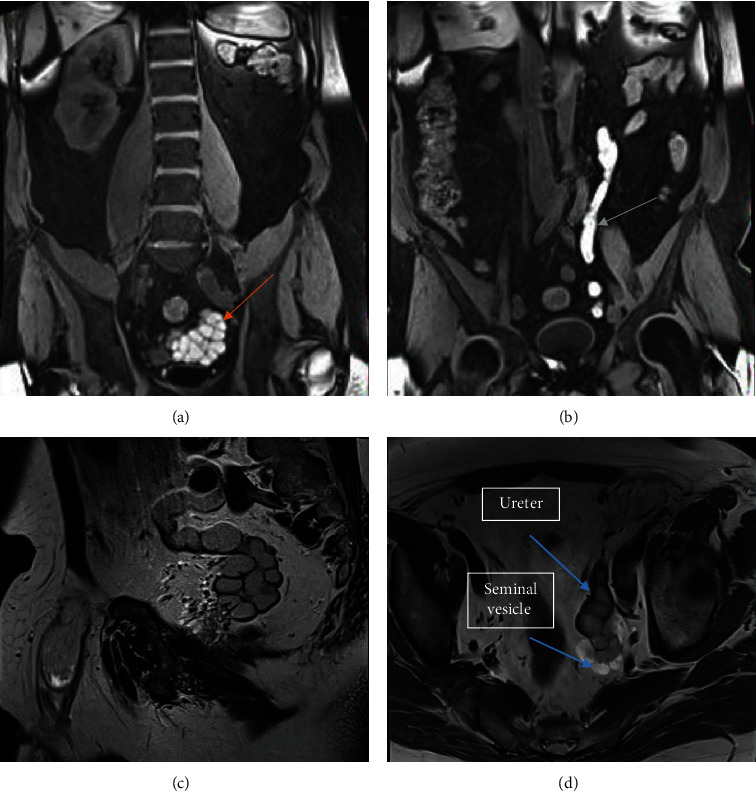
(a) MRI T1 coronal section showed left renal agenesis and dilatation of the left seminal vesicle (red arrow) that shows high signal content, suggesting hemorrhagic content or a high proteinaceous component; (b) tubular structure (green arrow) in the left lumbar and pelvic region that seemed to correspond to the dilated ureter (c). MRI T2 sagittal section reveals the ureter empties ectopically into the left seminal vesicle, which is markedly enlarged and multicystic and (d) shows in the axial the contrast enhancement difference between the ureter and seminal vesicle.

Abdominal-pelvic magnetic resonance imaging (MRI) was performed with acquisition of basal sequences, and after administration of intravenous gadolinium that revealed the absence of parenchyma of the left kidney, a tubular structure in the left lumbar and pelvic region was identified that seemed to correspond to the dilated ureter of up to 12 mm and with probable remnant of the renal pelvis in the most proximal lumbar portion. The ureter empties ectopically into the left seminal vesicle, which is markedly enlarged and multicystic in appearance, measuring approximately 6.9 cm in the oblique longitudinal major axis and 3.5 cm in the anteroposterior axis. Both the ureter and the seminal vesicle show high signal content in both T1 and T2, suggesting hemorrhagic content or a high proteinaceous component, and their walls and septa are thin, without significant contrast enhancement (Figure 2).

The left vas deferens is patent, being more thickened in its part closest to the seminal vesicle. Cystic dilatation of the left ejaculatory duct was observed.

Given the absence of symptoms related to this pathology, observation with periodic ultrasound is decided. The patient continues with his periodic revisions for the bladder tumor, without having any symptoms related to his Zinner syndrome.

## 3. Discussion

In general, seminal vesicle cysts are identified between the second and third decades of life, during the period of greatest sexual and reproductive activity [2].

There are around 200 published cases in the literature [2], but given the widespread use of imaging tests, it is suspected that there are more cases. A population-based screening study shows that the reported incidence of cystic lesions in the pelvis with agenesis or ipsilateral renal dysplasia is between 0.00035% and 0.0046%. According to several studies, up to 68% of all seminal vesicle cysts are associated with ipsilateral renal agenesis [4].

The especially remarkable characteristics of our case are the findings found in the imaging tests that show the ureteral remnant emptying into the ipsilateral seminal vesicle given the infrequency of this presentation.

Zinner syndrome is considered the male equivalent of the female Mayer-Rokitansky-Kuster-Hauser syndrome [5]. It usually presents as the triad described above, although it can also be associated with other genitourinary disorders such as ureterocele, hypospadias, and testicular, epididymal, or adrenal gland disorders [2]. In the literature, the most frequent symptoms include dysuria, abnormal urinary frequency, prostatitis, perineal or scrotal pain, epididymitis, and painful ejaculation [4].

In our case, the patient remains completely asymptomatic and the diagnosis of Zinner syndrome occurs incidentally in the context of medical monitoring for non-muscle-invasive bladder carcinoma.

Between the fourth and seventh weeks of embryogenesis, the ureter originates as a dorsal bud from the distal mesonephric duct, spreading dorsocranially to find and induce differentiation of the metanephric blastema, which will form the kidney. The mesonephric duct is differentiated in the appendix from the epididymis, paradidymis, epididymis, vas deferens, ejaculatory ducts, seminal vesicle, and hemitrigone. Normal kidney development depends on induction by the ureteric bud and mesonephric duct [6].

If the ureteral bud originates from a more cephalic portion of the mesonephric duct, the delay in absorption of the common distal portion of the mesonephric duct results in delayed absorption, as well as an opening of the ureteral bud in an ectopic location at the trigone level, bladder neck, urethra, or organs derived from the mesonephric duct, such as the ejaculatory duct, the vas deferens, or the seminal vesicle as in the case of our patient [6, 7].

Ultrasound is usually the first imaging test when this condition is suspected, since it is a quick and affordable modality. Thanks to it, we will be able to detect the absence of the renal unit, as well as the presence of a cystic mass in the pelvic region. Computed tomography (CT) and MRI, especially the latter, are capable of assessing more clearly the lesions and their anatomical characteristics [8].

Differential diagnosis should be made with other entities such as laterally located prostatic cysts, diverticulosis of the vas deferens, ectopic ureterocele, and abscesses. The presence of tumors in seminal vesicle cysts is extremely rare, with two cases of papillary adenocarcinoma reported in the literature. Ultrasound findings are those of an anechoic lesion or with the presence of internal echoes due to bleeding or infection, with a thick and irregular wall, occasionally with calcifications, in a retroprostatic location [3].

The management of this syndrome depends on the symptoms presented by the patient. In asymptomatic or slightly symptomatic cases like ours, observation may be chosen, while patients presenting symptoms will require surgical treatment, either transurethral, transrectal drainage, open surgery, or minimally invasive techniques, the latter being the one that has produced the best results regarding blood loss and hospital stay [9]. Another surgical indication may be infertility [6].

A recently published study has also proposed a new strategy for the analysis of seminal vesicle lesions in the diagnosis and treatment of Zinner syndrome, based on the morphology of the cysts. The study analyzed the size, content, possible calcifications, cyst wall structure, and possible solid lesions in association with cysts [10].

## 4. Conclusion

Zinner syndrome is a rare entity, with CT and MRI being the diagnostic methods of choice in these patients, given that they allow us a very precise anatomical definition, as well as the diagnosis of other associated malformations.

Surgical treatment is the gold standard in symptomatic patients, while in asymptomatic patients, observation may be chosen.

## References

[1] Zinner A. (1914). Ein fall von intravesikaler samenblasenzyste. *Wiener Medizinische Wochenschrift*.

[2] Pereira B. J., Sousa L., Azinhais P. (2009). Zinner’s syndrome: an up-to-date review of the literature based on a clinical case. *Andrologia*.

[3] Livingston L., Larsen C. R. (2000). Seminal vesicle cyst with ipsilateral renal agenesis. *AJR. American Journal of Roentgenology*.

[4] Hergan B., Fellner F. A., Akbari K. (2020). Incidental imaging findings suggesting Zinner syndrome in a young patient with pulmonary embolism: a case report. *Radiology Case Reports*.

[5] Khanduri S., Katyal G., Sharma H., Goyal A., Singh N., Yadav H. (2017). Unique Association of multiple seminal vesicle cysts with contralateral renal agenesis: a rare variant of Zinner syndrome. *Cureus*.

[6] Roehrborn C. G., Schneider H. J., Rugendorff E. W. (1986). Embryological and diagnostic aspects of seminal vesicle cysts associated with upper urinary tract malformation. *The Journal of Urology*.

[7] Grimaldo S. R., Chapa L. A. F., Galán M. J. J., Ríos B. N. I., Guardiola F. A. (2008). Quistes de vesículas seminales con agenesia renal ipsilateral. Presentación de 3 casos y revisión de la literatura. *Revista Mexicana de Urología*.

[8] Mehra S., Ranjan R., Garga U. C. (2016). Zinner syndrome--a rare developmental anomaly of the mesonephric duct diagnosed on magnetic resonance imaging. *Radiology Case Reports*.

[9] Kord E., Zisman A., Darawsha A. E., Dally N., Noh P. H., Neheman A. (2017). Minimally invasive approach for treatment of seminal vesicle cyst associated with ipsilateral renal agenesis. *Urologia Internationalis*.

[10] Tan Z., Li B., Zhang L. (2020). Classifying seminal vesicle cysts in the diagnosis and treatment of Zinner syndrome: a report of six cases and review of available literature. *Andrologia*.

